# Ethanol resistance in *Drosophila melanogaster* has increased in parallel cold‐adapted populations and shows a variable genetic architecture within and between populations

**DOI:** 10.1002/ece3.8228

**Published:** 2021-10-20

**Authors:** Quentin D. Sprengelmeyer, John E. Pool

**Affiliations:** ^1^ Laboratory of Genetics University of Wisconsin‐Madison Madison Wisconsin USA

**Keywords:** adaptive evolution, alcohol, *Drosophila melanogaster*, genetic architecture, genetic differentiation, local adaptation, quantitative trait locus mapping

## Abstract

Understanding the genetic properties of adaptive trait evolution is a fundamental crux of biological inquiry that links molecular processes to biological diversity. Important uncertainties persist regarding the genetic predictability of adaptive trait change, the role of standing variation, and whether adaptation tends to result in the fixation of favored variants. Here, we use the recurrent evolution of enhanced ethanol resistance in *Drosophila melanogaster* during this species’ worldwide expansion as a promising system to add to our understanding of the genetics of adaptation. We find that elevated ethanol resistance has evolved at least three times in different cooler regions of the species’ modern range—not only at high latitude but also in two African high‐altitude regions. Applying a bulk segregant mapping framework, we find that the genetic architecture of ethanol resistance evolution differs substantially not only between our three resistant populations, but also between two crosses involving the same European population. We then apply population genetic scans for local adaptation within our quantitative trait locus regions, and we find potential contributions of genes with annotated roles in spindle localization, membrane composition, sterol and alcohol metabolism, and other processes. We also apply simulation‐based analyses that confirm the variable genetic basis of ethanol resistance and hint at a moderately polygenic architecture. However, these simulations indicate that larger‐scale studies will be needed to more clearly quantify the genetic architecture of adaptive evolution and to firmly connect trait evolution to specific causative loci.

## Graphical abstract

We show that ethanol resistance in *Drosophila melanogaster* (a long‐standing model trait in evolutionary research) has increased not only in high‐latitude non‐African populations but also in high‐altitude regions of Africa. We use bulk segregant QTL mapping to illustrate a variable genetic basis of adaptive trait evolution within‐ and between‐resistant populations with a moderately polygenic architecture.

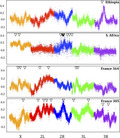

## 1 INTRODUCTION

The genetic basis of adaptive trait evolution is an area of great interest to biologists and has raised several key questions. There are two questions that are of particular interest to this study. For example, how polygenic is trait evolution (Wellenreuther & Hansson, 2016)? And do favored variants tend to reach fixation, or stop rising because selective pressures change or traits reach their new optima (Thornton, 2019)?

Early theory suggested that adaptive trait evolution is the result of many genes with small effect (Fisher, 1930), or mutations with intermediate effect size (Kimura, 1983). A more recent hypothesis proposes that depending on where a population is relative to the phenotypic optimum will dictate whether few mutations with large effect or many small effect mutations will be favored (Orr, 1998). This model argues that when an organism first encounters a novel environment, genes of large effect size would be most abundant and as the population moves closer to an optimal phenotype, the effect size would decrease, with an overall geometric distribution of effect sizes predicted. Alternatively, migration–selection balance may favor larger effect sizes underlying local adaptation (Yeaman & Whitlock, 2011), whereas an important role for previously deleterious standing variation may lead to a greater role for smaller effects instead (Dittmar et al., 2016).

There have been many studies that investigate the genetic architecture of novel traits. A classic example is the body color of peppered moths found in England. It was discovered that a single gene is responsible for the light and dark morph (van’t Hof et al., 2011). While a number of other cases of a simple genetic basis to trait evolution are known, it is unclear how common they are in light of ascertainment bias (Rockman, 2012). As a contrasting example, studies that have looked at human height differences between populations found that this trait may be due to many genes (Gudbjartsson et al., 2008; Turchin et al., 2012). However, recent studies have called into question these results and suggest that population stratification has led to overestimating the number of genes involved (Berg et al., 2019; Sohail et al., 2019). There is growing evidence for polygenic adaptation underlying trait evolution (Barghi et al., 2020). However, the term “polygenic adaptation” actually encompasses a broad array of possible evolutionary scenarios in terms of the number of loci involved, the magnitudes of their effects on evolving traits, and their frequencies before and after being selectively favored.

Theoretic and empirical studies have led to testable predictions about polygenic adaptation. First, depending in part on the number and effect sizes of beneficial alleles, they may not always reach fixation (Barghi et al., 2020; Barghi & Schlötterer, 2020; Höllinger et al., 2019; John and Stephan, 2020; Stephan, 2016; Thornton, 2019). Experimental evolution studies, including in *Drosophila*, have begun to provide evidence for genetically heterogeneous responses to selection. Considerable variability among replicates in the loci that respond to selection on environmental tolerances (Barghi et al., 2019; Griffin et al., 2017) suggests an abundance of segregating variation in natural populations for selection in natural populations to act upon, not all of which is needed for individuals to display an evolved trait. And when another study selected for viral resistance, plateaus in the frequencies of initially favored alleles were observed (Faria et al., 2018). If similar dynamics underlie natural instances of trait evolution, then different individuals from the same population may often have distinct genetic architectures underlying the same evolved trait. As one example, a previous study found that melanism had a variable genetic basis within three different highland African populations of *D*. *melanogaster* (Bastide et al., 2016).

*Drosophila melanogaster* originated in woodland environments of southern‐central Africa and then expanded throughout Africa beginning ~13,000 years ago (Sprengelmeyer et al., 2020). The species appears to have crossed the Sahara relatively soon after their expansion started and may have only reached Europe ~1800 years ago. During the migration out of their ancestral habitat, populations of *D*. *melanogaster* encountered many novel environments, which included equatorial tropical rainforest, northern temperate grassland, and high‐altitude alpine regions. Each of these different ecosystems provides unique selection pressures that may have forced local populations to acquire novel traits in order to survive. For example, increased ultraviolet radiation found at higher altitudes might have caused populations to evolve darker cuticle pigmentation (Bastide et al., 2014). And populations at high latitude and altitude have independently evolved elevated cold tolerance (Pool et al., 2017).

Ethanol resistance is another trait that has evolved in *D*. *melanogaster*. When compared to its sister species *D*. *simulans*, *D*. *melanogaster* are more ethanol resistant (McKenzie & Parsons, 1972). Within *D*. *melanogaster*, ethanol resistance has shown a positive correlation with latitude (Cohan & Graf, 1985; David & Bocquet, 1975) with populations living in breweries and wine cellars of France and Spain being the most resistant (McKenzie & Parsons, 1972; Merçot et al., 1994). Female flies lay their eggs on ethanol‐producing fermenting fruit and having a higher ethanol resistance may provide more available resources (Merçot et al., 1994). There is evidence that *D*. *melanogaster* prefers to lay their eggs on medium that contains alcohol (McKenzie & Parsons, 1972), which can be a defense against parasitoids (Kacsoh et al., 2013; Milan et al., 2012).

Alcohol metabolism in *D*. *melanogaster* involves ethanol being converted to acetaldehyde by *ADH* (Greer et al., 1993). *ADH* and *ALDH* convert acetaldehyde to acetate. Acetate can be turned into acetyl‐CoA, which can be used in the production of fatty acids, the citric acid cycle, and other pathways. Differences at the *Adh* gene are correlated with improved alcohol resistance (David & Bocquet, 1976), with the “fast” allele having a higher resistance compared with the “slow” allele. David and Bocquet (1975) found a latitudinal gradient and populations at higher latitudes tend to be more resistant and also have a higher *Adh*_fast_ frequency. However, *D*. *funebris*, *D*. *littoralis*, and *D*. *mercatorum* all display ethanol resistance but low ADH activity, whereas in spite of high ADH activity, *D*. *ercepeae* are classified as being sensitive to alcohol (Merçot et al., 1994). It has been hypothesized that the *Adh*_fast_ and *Adh*_slow_ polymorphism has been maintained by a temperature dependent balancing selection (Van Delden et al., 1978). However, Siddiq and Thornton (2019) found *Adh*_fast_ protein is neither less stable nor active at high temperatures and will increase ethanol resistance along with survivorship at all temperatures. Further, when they analyzed a population genomic data set, there was not a signature of balancing selection in the *Adh* gene.

Changes at *ALDH* can also increase ethanol resistance (Fry & Saweikis, 2006). Fry et al. (2008) also showed that there is an amino acid difference between more resistant populations found in higher latitudes and less resistant flies found in lower latitudes. It has also been found that European flies can have higher *ALDH* enzyme activity compared with less resistant African flies even without the amino acid polymorphism (Fry, 2014). Chakraborty and Fry (2016) found that polymorphisms in *ALDH* are maintained by environmental conditions, and their transgenic experiments confirmed effects on lifetime fitness on ethanol‐supplemented medium specifically.

Although *ADH* and *ALDH* play an important role, they are not the only genes involved in ethanol resistance. Other genes linked to ethanol resistance encompass a wide range of functions such as lipid membrane physiology (Montooth et al., 2006), ion channels (Cowmeadow et al., 2005), central nervous system (Chandler et al., 1998), zinc retention (Zhao et al., 2009), and feeding behavior and behavioral responses to ethanol (Fochler et al., 2017). Signor and Nuzhdin (2018) found that many genes display plasticity in expression and splicing in response to ethanol exposure. Other studies that focused on changes in gene expression (Morozova et al., 2006) or histone modification (Ghezzi et al., 2013) have also found that numerous genes respond to ethanol exposure.

In the present study, we use ethanol resistance in *D*. *melanogaster* to further our understanding of the genetic architecture of adaptive trait evolution. Although a number of prior studies have studied variation in ethanol resistance both within and between *D*. *melanogaster* populations (as indicated above), the present study integrates resources and approaches not previously deployed in this pursuit. We use wild populations from their ancestral range (Zambia), along with multiple populations that display elevated ethanol resistance: from high altitude sub‐Saharan Africa (Ethiopia and South Africa) and from high latitude (France). We note that each of the ethanol‐resistant populations has also evolved elevated cold tolerance, and in light of the species’ expansion history, this trait change is thought to have arisen independently in each of these three populations’ histories (Pool et al., 2017). To detect QTLs that are involved in this adaptive trait evolution, we perform bulk segregant analysis (Pool, 2016). We then leverage population genomic data to identify signatures of local adaptation within QTLs, while also applying Gene Ontology (GO) enrichment and genotype–phenotype association testing to search for potential causative genes. We also perform simulations to explore the parameters (number of detected QTLs, environmental variance, and QTL strength) involved in the genetic architecture of this adaptive trait change.

## 2 MATERIALS AND METHODS

### 2.1 Experimental populations

All flies used in the experiment had been inbred for 8 generations from wild‐caught isofemale lines (Lack et al., 2015). The sub‐Saharan African populations came from Fiche, Ethiopia (EF, 9.81°N, 38.63°E, alt. 3070 m), Dullstroom, South Africa (SD, 25.42°S, 30.10°E, alt. 2000 m), and Siavonga, Zambia (ZI, 16.54°S, 28.72°E, alt. 530 m). The French samples are from Lyon, France (FR, 45.77°N, 4.86°E, alt. 175 m). Flies were all raised at 20°C on medium prepared in batches of 4.5 L water, 500 ml cornmeal, 500 ml molasses, 200 ml yeast, 54 g agar, 20 ml propionic acid, and 45 ml tegosept 10% (in 95% ethanol).

### 2.2 Ethanol resistance

To test for population differences in ethanol resistance, we measured mobility over a 6‐h period. We collected data from the offspring of inbred lines from the same population. The number of flies and pairs of lines used was as follows: FR: 10 lines, *N* = 300, EF: 10 lines, *N* = 300, SD: 10 lines, *N* = 300 and ZI: 10 lines, *N* = 300. We then placed 3‐ to 5‐day‐old female flies into 50‐ml falcon tubes with a single tissue placed in the bottom that was saturated with 1.5 ml of 3% sucrose (molasses) solution that contained 8% ethanol (Fry, 2014). Every 15 min, we visually checked tubes and scored flies that did not move after the vial was flicked as “immobile.”

To test for evidence that local adaptation has influenced population differences in ethanol resistance, we calculated *Q*_ST_ values (Lande, 1992; Miller et al., 2008; Spitze, 1993) based on two‐population comparisons of the above phenotypic data. We compared these *Q*_ST_ values against *F*_ST_ values from short windows based on population genomic data from the same population samples available from the *Drosophila* Genome Nexus (Lack et al., 2016). For *F*_ST_ calculations, we used the same number of inbred genomes that were phenotyped (*n* = 10) and used diversity‐scaled windows that averaged ~700 bp in length (specifically, based on 20 non‐singleton SNPs from the full Zambia ZI sample). *F*_ST_ for individual short windows should have a conservatively higher neutral variance than *Q*_ST_ for a polygenic trait.

### 2.3 Bulk segregant analysis

To ascertain areas of local adaptation responsible for higher ethanol resistance, bulk segregant analysis was performed to detect quantitative trait loci (QTL) (Pool, 2016). Population cages were started from reciprocal crosses between eight inbred parental individuals of low resistant (Zambia) and one each of the more resistant African populations (Ethiopia and South Africa) lines and strains from two French populations (Table S1). From each reciprocal cross, 125 F1 offspring of each sex were used to establish the second generation. These mapping populations for the rest of the (non‐overlapping) generations were maintained at ~1200 individuals. The flies were housed in 28 × 14 × 15 cm plastic cages that contain 14 vials with a medium that contains molasses, cornmeal, yeast, agar, and antimicrobial agents at ~20°C. Adult flies were allowed to lay eggs on the food for one week before being removed. The food vials were replaced when adult flies in the cage were 7–10 days old. At the 15th generation, 600 3‐ to 5‐day‐old female flies from each population cage were exposed to the 8% ethanol mobility assay described above. Twelve batches of 50 flies were simultaneously placed into twelve inverted 50‐ml falcon tubes containing a Kimwipe (resting on the cap) saturated with 1.5 ml of an aqueous solution containing 8% ethanol and 3% molasses. When flies became immobile, they were collected by removing the bottom cap. The cap was then replaced with a new cap, with a Kimwipe containing the same solution. Female flies were placed into two pools to be sequenced, the 10% least resistant (*N* = 60) representing the first flies to lose mobility, and the 10% most resistant (*N* = 60) representing the last flies still mobile. The remaining flies were discarded.

### 2.4 Genome preparation

We sequenced the genomes of pooled samples (*N* = 30 individuals) for the parental lines and two such pools for each of the low and high resistant groups (0%–5% and 5%–10% extremes for each direction, summing to *N* = 60 total for each extreme). Genomic DNA was obtained using a chloroform extraction and ethanol precipitation protocol. The DNA was fragmented with a Bioruptor sonicator (Diagenode) and paired‐end libraries with ~300 bp inserts prepared using NEBNext DNA Library Prep Reagent Set for Illumina (New England Biolabs no. E6000L). Each library's concentration and quality was analyzed with an Agilent 2100 Bioanalyzer (Agilent Technologies, Inc.). The prepared libraries were sequenced at UW‐Madison Biotechnology Center on the Illumina HiSeq 2000 platform. Having concluded that the full 10% extremes would best be analyzed together (Pool, 2016), we merged reads from the 0%–5% and 5%–10% pools (similar numbers of reads were obtained from these pools in each case) before proceeding with the analysis.

### 2.5 Genome alignment

All the raw data that passed the Illumina filters were processed using a Perl‐scripted pipeline. Reads from each sequenced genome were mapped to the *D*. *melanogaster* reference genome (release 5.57) obtained from Flybase (www.flybase.org), with the default parameters in BWA ver. 0.6.2‐r126 (Li & Durbin, 2009). Using Stampy ver. 1.0.21 (Lunter & Goodson, 2011), the BAM files were then remapped in order to mirror the *Drosophila* Genome Nexus pipeline used for the parental strain genomes (Lack et al., 2015). With samtools ver. 0.1.18 (Li et al., 2009), reads were filtered for a mapping quality of 20 and for proper pairs. The BAM files were further processed by removing unmapped reads and sorted by coordinate, and PCR duplicates were marked using Picard ver. 1.109 (http://picard.sourceforge.net). To improve the alignment around indels, we used GATK ver. 3.2 (McKenna et al., 2010). The average depth of coverage per genome was calculated for the parental lines and the low and high resistance lines (Table S1).

### 2.6 Quantitative trait locus mapping

The PoPoolation2 ver. 1.201 software package (Kofler et al., 2011) was used to create synchronized mpileup files for the aligned genomes. For each biallelic SNP, an ancestry difference (*a_d_*) was calculated (Bastide et al., 2016), which was calculated as:

(1) $$a_{d}=\left(f_{H}‐f_{L}\right)/\left(p_{H}‐p_{L}\right)$$

where *p_H_* is the frequency of parental high resistant allele, *p_L_* is the low resistant parental allele, *f_H_* is the frequency of parental high resistant allele in the F12 offspring, and *f_L_* is the frequency of parental low resistant allele F12 offspring. The five analyzed chromosomal arms (X, 2L, 2R, 3L, and 3R) were divided into windows based on 200 non‐singleton SNPs in the Zambia population (Lack et al., 2015), which created 2728, 3131, 2357, 2956, and 2935 windows, respectively, each roughly 8.4‐kb in size on average. Only sites that had a parental allele frequency difference of ≥0.25 were used in the analysis. A simulation‐based inference for BSA mapping (SIBSAM) was performed (Pool, 2016) to identify significant QTLs and calculate their confidence intervals and effect sizes. The custom scripts used for SIBSAM can be found at: http://github.com/JohnEPool/SIBSAM1. SIBSAM is able to evaluate both primary QTL peaks and flanking secondary QTL peaks, evaluating whether ragged peaks contain significant evidence for more than one QTL. Forward simulations incorporate recombination in multiple individuals for multiple generations, selection on phenotype in the final generation with additivity, plus environmental variance, and then the sampling of sequence reads to obtain *a_d_*.

### 2.7 Genetic differentiation and Gene Ontology enrichment analysis

To find evidence of local adaptation and produce a list of candidate genes found within the significant QTLs, window *F*_ST,_ and maximum SNP *F*_ST_ per window (hereafter “SNP *F*_ST_”), and the haplotype statistic *χ*_MD_ (Lange & Pool, 2016) were analyzed. Pairwise comparisons were made between the low ethanol‐resistant population Zambia and each of the high resistance populations. Genomes from Zambia (*n* = 197), South Africa (*n* = 61), Ethiopia (*n* = 68), and France (*n* = 96) were used from the *Drosophila* Genome Nexus (Lack et al., 2015). The *χ*_MD_ compares length of identical haplotype blocks among individuals in one population versus another. The comparisons were made within each of the five chromosomal arms (X, 2L, 2R, 3L, and 3R), which were divided into windows based on SNP density (Lack et al., 2015). The idea behind *χ*_MD_ is that in a recently selected population, longer stretches of identical haplotypes will not have had time for recombination or mutation to break up longer identical tracts. A chromosomal arm quantile outlier approach was used to focus on genes with an extreme population genetic signal. Only windows that were in the top 2.5% quantile in any of the three statistics were classified as outliers. To form an outlier region, a maximum of two non‐outlier windows were allowed between two outlier windows. Genes associated with outlier windows (overlapping them or the nearest gene in either direction) were retained for subsequent analysis. The outlier genes identified in significant QTL regions were used for window‐based gene ontology (GO) enrichment analysis (as implemented in Pool et al., 2012) to identify functional categories that differ between low and high resistance populations. A *p* value was calculated based on the probability of observing a given number of outlier genes from a GO category. *p* values were obtained from permutation in which outlier region was randomly reassigned 10,000 times.

### 2.8 Genotype–phenotype association testing

Phenotypic data were collected on 51 France inbred strains with sequenced genomes from the Drosophila Genome Nexus genomic resource (Lack et al., 2016). Here, we followed the ethanol assay described above, except that 50 flies from each strain were placed in a single falcon tube and the immobilization times for each individual were recorded (and subsequently averaged for the strain). To capture the variation in ethanol resistance found in the France population, the ethanol concentration used was 18%. The assay was otherwise identical to the inter‐population assay described above. Genotype–phenotype associations were analyzed with the R package rrBLUP version 3.1 (Endelman, 2011). Only regions within the QTL peaks of <2 Mb in length in the two France crosses were examined. Within these peaks, only SNPs that had a called allele of >25% and a minor allele frequency >5% among the 51 France strains were analyzed. One thousand permutations of the phenotypic data were used to calculate the significant threshold.

### 2.9 Simulations of genetic architecture and association testing power

We performed simulations to better understand the genetic architecture of this adaptive trait, using modified versions of SIBSAM scripts. These simulations involved three steps. First, we calibrated the number and strengths of QTLs to match the empirical data from the two France/Zambia crosses. To do this, we analyzed a range of values for three different parameters: (1) the number of detected QTLs (10, 20, 30, 40, and 50); (2) environmental variance, how much of the phenotypic trait is caused by factors other than genetic factors (0.5, 0.6, 0.7, 0.8, and 0.9); and (3) QTL strength. Here, we used a gamma distribution (shape parameter 0.5, 1, 2, 4, and 8, and scale parameter fixed at 1 because it is not relevant in this relative context). We performed 10,000 simulation replicates for all parameter combinations for both France crosses.

For these comparisons between empirical and simulated QTL mapping data, we used a simplified set of QTL criteria in order to avoid the computational infeasible requirement of running full SIBSAM inference to identify significant QTLs from each simulated replicate. Specifically, we defined QTLs as having ancestry difference greater than 0.16. The flanking secondary QTL peaks were defined as having a secondary deviation (the magnitude of ancestry difference recovery from a local valley; Pool, 2016) greater than 0.16. These criteria were chosen to largely recapitulate the same QTLs found to be significant from the empirical data.

We then looked at four summary statistics: the mean ancestry difference and its standard deviation across all windows, the mean QTL peak height, and the number of QTLs. We calculated the relative error sum of all the replicates for each combination using the empirical values: mean peak height 0.256, number of QTLs 18, mean ancestry difference 0.041, and standard deviation 0.083. The parameter combination with the lowest mean relative error sum was then used to perform the next step to calibrate the frequency of all QTLs. To analyze how well the top model performed, we performed bootstrapping among the 10,000 replicate simulations from both the top model and one of the other 125 parameter combinations, monitoring the proportion of 10,000 bootstrap replicates in which the top model still had a lower error.

Next, we wanted to see which QTL frequency along with fixed parameters from the previous step would match the proportion of empirical QTL peaks overlapping between the two crosses. We ran 10,000 replicates of each of the different frequency values: 0.05, 0.10, 0.15, 0.20, 0.25, 0.30, 0.35, 0.40, 0.45, and 0.50—where these values indicated the probability that a given QTL in one cross would also be present in the second cross. Each QTL was considered to overlap if its peak fell within a simplified “QTL region” from another cross (defined as the area in which a peak exceeds an ancestry difference or secondary deviation of 0.16).

Finally, we estimated association testing power for different scenarios involved with sample sizes and allele frequencies. We used sample sizes of 50, 100, 200, 500, and 1000 and allele frequencies of 5%, 10%, 20%, 30%, 40%, and 50%. For each parameter combination, we created genotypes and phenotypes. Genotypes were assigned by first determining if the individual was either homozygous or heterozygous based on empirical residual heterozygosity levels of 35% in the France population (Lack et al., 2016). If an individual was homozygous, then they had one draw of getting either the ancestral or derived allele and it was added twice. If an individual was heterozygous, then they had two independent draws of getting either the ancestral or derived allele. We translated the QTL frequency identified in the previous into allele frequency using the equation:

(2) $$0.65q+0.35\left(q2+2q\left(1‐q\right)\right)$$

where 0.65 is the frequency of being homozygous and 0.35 is the frequency of being heterozygous and *q* is the frequency of the derived allele. Once the genotypes were established, the phenotypic trait values could be assigned. If the individual genotype at the locus was homozygous for the derived allele, then the full QTL strength was added. If the genotype was heterozygous, then half the QTL strength was added, and if the genotype was homozygous for the ancestral allele, then nothing was added to the trait value (i.e., assuming additivity). In light of the replicated phenotyping of individuals from each inbred line, no environmental variance was simulated. We performed 1000 simulated genotype–phenotype association replicates for each parameter combination and recorded the proportion of total alleles that exceeded the empirical permutation ‐log(*P*) threshold of 6.17.

## 3 RESULTS

### 3.1 Population differences in ethanol resistance

**FIGURE 1:**
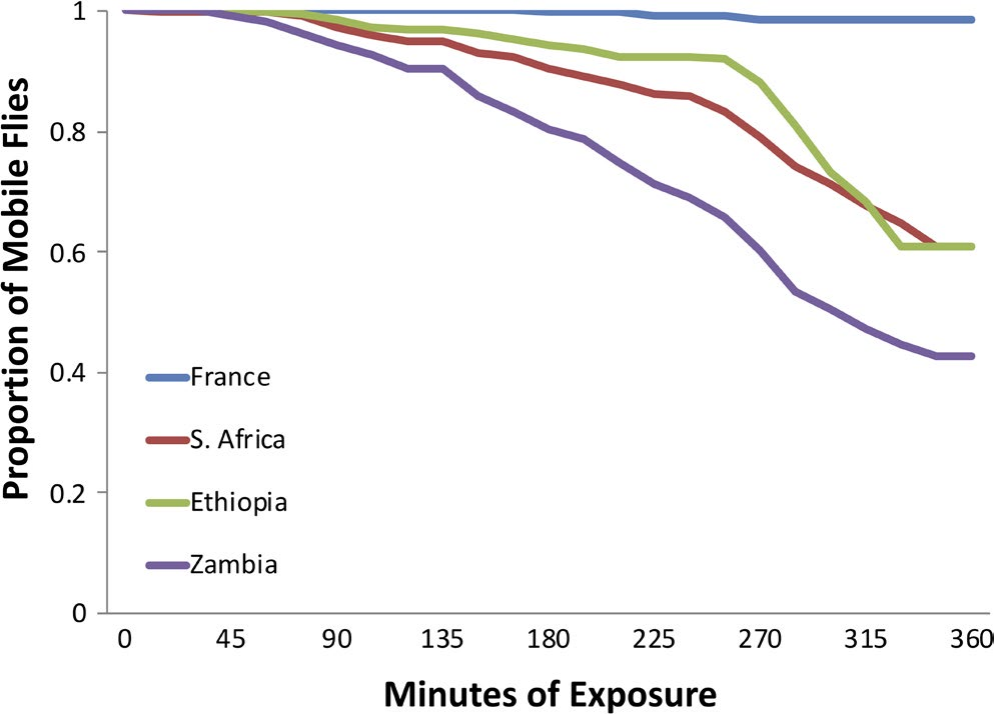
*Drosophila melanogaster* populations differ strongly in their resistance to concentrated ethanol vapor. The percentage of flies that remained mobile after being exposed to 8% ethanol is shown across a 6‐h interval. Here, Zambia represents a population from the ancestral range of the species, South Africa and Ethiopia derive from relatively cooler high elevation locations, and France represents a cooler temperate latitude

We performed a phenotypic assay of adult exposure to 8% ethanol vapor, using flies from ten independent inbred strains from each of four population samples. This assay revealed variation in ethanol resistance among the populations studied (Figure 1). As expected, the France population (David et al., 1986) had the highest resistance with only 1% of the individuals immobile after six hours of exposure (mean 379.5 min of mobility, SD 16.7). The Zambia population from the ancestral range had the lowest resistance; after six hours, there was 57% immobility (mean 267.4 min, SD 93.5). The two high altitude African populations, Ethiopia and South Africa, were not as resistant as the France population, but more resistant than Zambia. Both South Africa (mean 307.5 min, SD 78.5) and Ethiopia (mean 318.5 min, SD 63.7) had ~40% of the individuals immobile at 6 h. We performed an individual‐based ANOVA between each of the more resistant population (Ethiopia, S. Africa, and France) and the least resistant Zambian population, using the 6‐h data. We found that in spite of substantial within‐population variation among strains, the three former populations were each significantly more resistant than the Zambian population (*p* ≤ 3.21 × 10^−11^ in each case; Table S2).

We used *Q*_ST_ values (Lande, 1992; Miller et al., 2008; Spitze, 1993) to quantify phenotypic differentiation between the three ethanol‐resistant populations and the least tolerant Zambian population. For all three comparisons, the *Q*_ST_ values were much greater than the *F*_ST_ values. The France–Zambia comparison had a *Q*_ST_ value of 0.548 compared with a mean *F*_ST_ value of 0.226, with 0.015% of genomic windows having an *F*_ST_ >*Q*_ST_. The Ethiopia–Zambia comparison had a *Q*_ST_ value of 0.216 compared with *F*_ST_ value of 0.137, with 0.16% of genomic windows having an *F*_ST_ greater than *Q*_ST_. The South Africa–Zambia comparison had a *Q*_ST_ value of 0.115 compared with *F*_ST_ value of 0.024, with 0.027% of genomic windows have an *F*_ST_ greater than *Q*_ST_. If genetic differentiation across most of the genome is primarily the result of neutral processes, then high values of *F*_ST_ or *Q*_ST_ that fall outside the genomic distribution of *F*_ST_ may reflect local adaptation rather than population history alone. Given that ethanol resistance *Q*_ST_ values are indeed greater than the vast majority of *F*_ST_ values, it appears that this trait has evolved under the influence of local adaptation, acting either upon this trait directly or upon pleiotropically correlated traits.

### 3.2 Quantitative trait locus mapping

**FIGURE 2:**
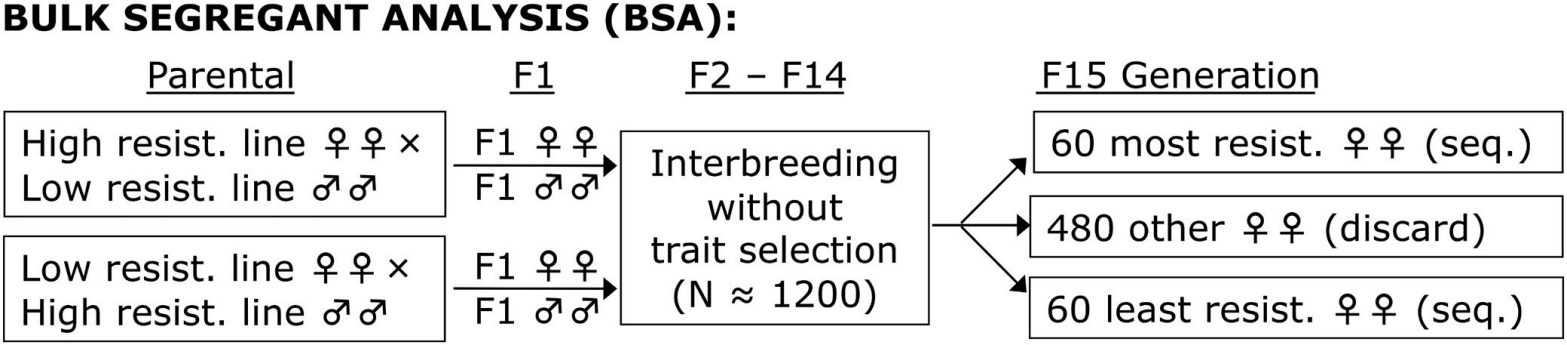
The bulk QTL mapping experimental design is illustrated. Population cages were started from reciprocal crosses between eight inbred parental individuals from a single low‐resistance Zambia strain and an equal number of individuals from a single strain from one of the more resistant populations (Ethiopia, France, or South Africa). 125 F1 offspring from each reciprocal cross were combined, and the mapping population was allowed to interbreed with a population size of roughly 1200 until the 15th generation. At that point, females were tested for ethanol resistance, and the first 10% and last 10% to become immobile were isolated for pooled sequencing

We performed QTL mapping using four different between‐population crosses using individual inbred strains, each of which involved the low‐resistance Zambia population. Of the higher resistance parental strains, two were independent France strains, and one each was from the African high altitude populations, Ethiopia and South Africa. We allowed offspring of reciprocal crosses to interbreed without selection at a fairly large population size (*N* ≈ 1200) until the 15th generation, at which time 600 adult females were exposed to ethanol vapor and the top and bottom 10% of individuals were isolated and subject to pooled genomic sequencing (Figure 2; *Materials and Methods*). Primary and secondary QTL peaks, along with their estimated effect sizes and genomic confidence intervals, were then identified using SIBSAM (Pool, 2016).

**FIGURE 3:**
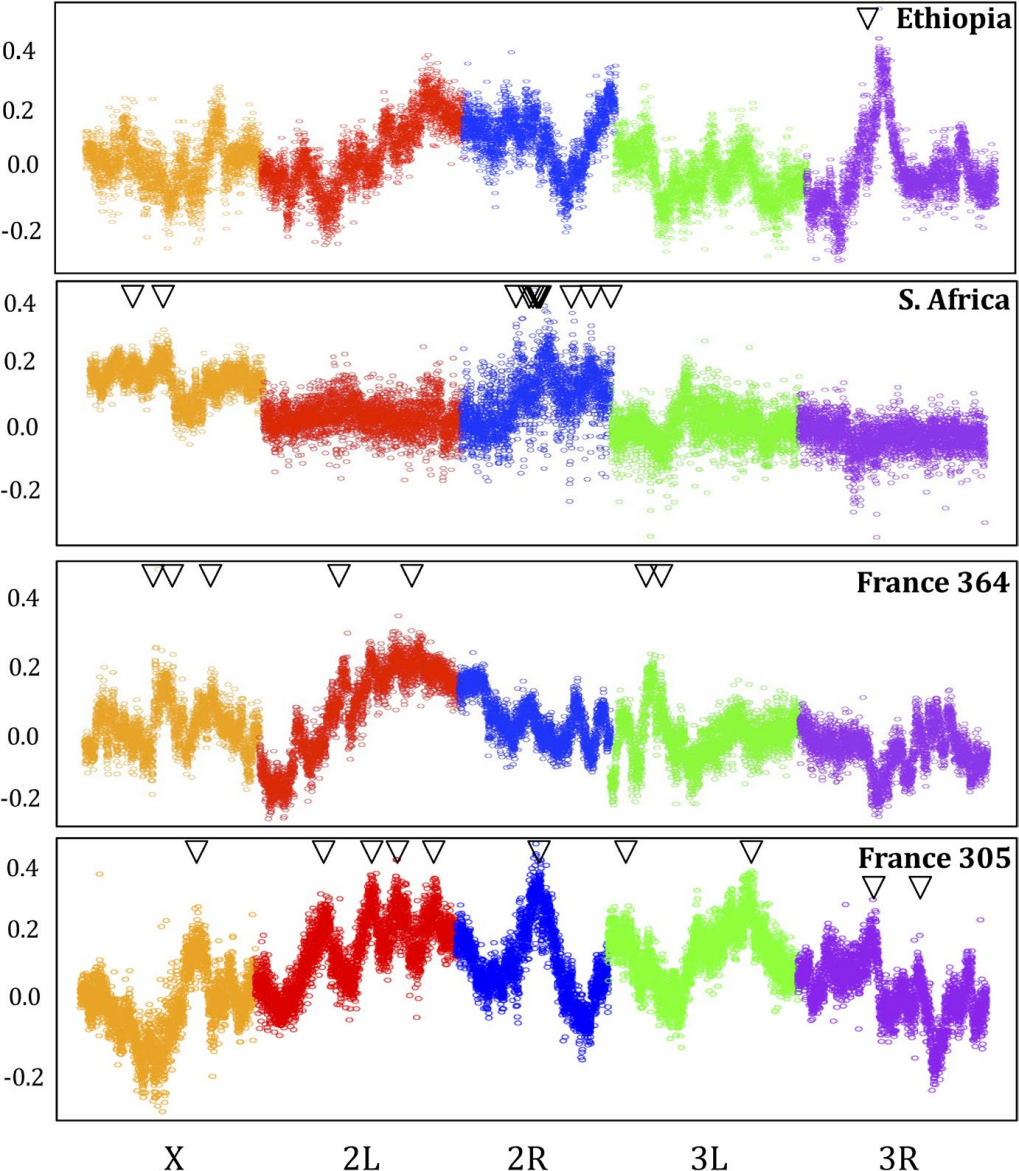
Significant QTL peaks for Ethiopia, South Africa, and France crosses are shown, based on bulk segregant mapping from 15th generation cross offspring. A point for each ~8 kb window corresponds to the average difference in the frequency of the resistant parental strain’s allele between the high‐ and low‐resistance F15 pools (i.e., “ancestry difference,” *y*‐axis). Significant QTLs are denoted with an arrow. The South Africa cross includes a total of 10 significant QTLs on chromosome arm 2R. The significance threshold for primary peaks is approximately 0.16

The four mapping crosses revealed a total of 32 significant peaks (Figure 3; Table S3). Whereas the Ethiopia cross had just three significant QTLs with estimated effect sizes between 15% and 20%, the South Africa cross had a total of 12 significant peaks, ten of which were on chromosome arm 2R and two on the X chromosome, and these 12 QTLs had estimated effects sizes between 7% and 13%. Between the two France crosses there were 17 peaks, ten from the cross involving strain FR305N and seven for FR364N, which collectively ranged in estimated effect size from 6% to 27%. Encouragingly, the highest peaks in each cross were estimated to have narrow genomic confidence intervals (Table S3). We note that under some scenarios, effect size may be overestimated (Pool, 2016), and consistent with this possibility, the point estimates of QTL effect sizes sum to more than 100% for three out of four crosses (Table S3).

**FIGURE 4:**
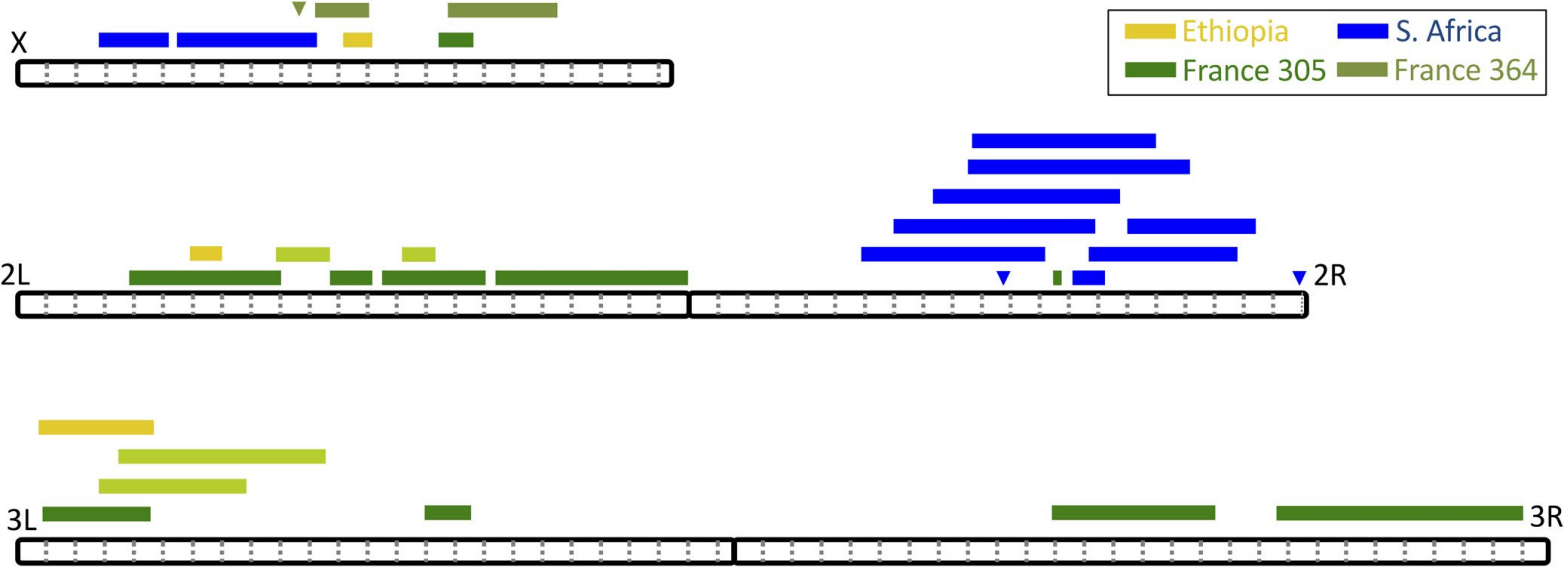
QTL locations vary within and between ethanol‐resistant populations. The locations of significant QTLs on the five euchromatic chromosome arms of *D*. *melanogaster* are shown. The colors indicate ethanol resistance mapping crosses involving Ethiopia, South Africa, and France lines 305 and 364. The width of each box indicates the 90% C.I. of each QTL. Intervals that are <10 kb in width are marked with triangles. Dotted gray lines indicate Mb increments

Overlap between QTL peaks may occur by chance or due to a shared genetic basis of ethanol resistance differences between crosses. Between the two France crosses, there were six regions where QTL peaks overlapped with genomic confidence intervals involving a total of 6 out of the 17 QTLs (Figure 4). In a few cases, overlapping QTLs were found between crosses from different populations. Ethiopia shared two distinct QTLs with each of the France crosses, while South Africa shared one QTL with FR364N. The two high‐altitude populations, Ethiopia and South Africa, did not share any peaks. Hence, while there is some unconfirmed potential for genetic parallelism between ethanol resistance in different *D*. *melanogaster* populations, most QTLs tend to be unique between a given pair of crosses—even when two crosses involve the same France and Zambia populations. While chance false‐positive and false‐negative results may contribute to differences in QTL detection, distinct genetic paths to ethanol resistance in different populations, as well as genetic heterogeneity in the architecture of ethanol resistance within populations, may contribute to these results as well, as further explored below.

### 3.3 Potential targets of local adaptation within QTL regions

Strong differences in genetic variation between the least resistant Zambia population and one of the more resistant Ethiopia, South Africa, and France populations may signify genes subject to local adaptation, and some of these signals could relate to the trait in question. Therefore, to identify possible candidate genes for ethanol resistance evolution within the significant QTLs, we used three population genetic statistics, window *F*_ST_, maximum SNP *F*_ST_ within a window, and the haplotype statistic *χ*_MD_ (Table S4). These statistics may have differing power to detect local adaptation depending on whether selective sweeps are complete or incomplete, or hard versus soft (Lange & Pool, 2016). We used a quantile approach focusing on regions that had one of the three statistics with a quantile below 0.025 (Table S5). This analysis yielded both genes with known functions that may relate to our trait, and genes with no such known functions. While any of these genes might contribute to ethanol resistance evolution, we mention below a few plausible candidates.

Within the South Africa QTLs, peaks on chromosome arms X and 2R each have one outlier redox gene, *Pp2C1* and *Nox*, respectively. Genes involved in regulating oxidative stress have previously been implicated in *Drosophila* ethanol resistance (Awofala et al., 2012). Of potential relevance in light of our aerosol ethanol assay, several genes involved in the development of chitin also have population genetic signals: *ovo*, *mgl*, and *CG1367*. Potential candidate genes found in Ethiopia QTLs include the following: *Shab* and *Teh2* (ion channels), and *m* (cuticle development).

Notably in one of the France crosses, *Adh* was identified as a population genetic outlier in a QTL region. We note that the Fast allele associated with greater ethanol resistance is at a 88% frequency in our France population sample, compared to 0% in Zambia. Other genes found in one of the two France crosses included several potentially involved in alcohol metabolism (*CG5065*, *CG6650*, *CG8303*, *CG9521*, *CG13091*, *CG15601*, *CG43658*, *Pis*), as well as ion channels (*para*, *ppk*, *sh*) and other genes involved in neurotransmission (*be*, *CG33639*). Diverse aspects of nervous system function have previously been linked to alcohol resistance (Morozova et al., 2015; Park et al., 2017).

Between the two France crosses, shared candidate genes included the following: *CG45065* (alcohol metabolism), *CG9503* (choline/aldehyde metabolism), *bgm* and *pgdy* (fatty acid metabolism), *hiw* (synapse organization), and *eag* (ion channel, response to ether). South Africa and FR364N had two candidate genes of interest, *CG32698* (carbonate dehydratase) and *CG1986* (lipase). Lipid levels are known to influence ethanol resistance (Geer et al., 1991; Lieber & Savolainen, 1984).

### 3.4 Gene Ontology enrichment

While local adaptation outliers within QTLs represent promising candidates for causative loci, our suspicion would be strengthened if particular types of genes recurrently appeared in the intersection of our mapping and population genetic analyses. Therefore, as a hypothesis‐generating exercise, we conducted a GO enrichment analysis on the set of genes both located within a QTL region from any of our crosses and also associated with a population genetic outlier region for that same resistant population. Alcohol metabolism genes were enriched in this analysis (*p* = .00356; Table S6). The categories showing the strongest enrichment (*p* values below 0.001) corresponded to functions previously linked to ethanol response: spindle localization (Hass et al., 2019), sterol biosynthesis (Mo et al., 2019; Stanley et al., 2010), and microvillus membrane (Bjorkman & Jessop, 1994). Other enriched categories related to the perception of sound and light, cuticle development, response to hypoxia, histone H4 acetylation (Ghezzi et al., 2013), and zinc transport (Zhao et al., 2009).

### 3.5 Genotype–phenotype association testing

Given that the genetic architecture of ethanol resistance in the France population appears to be genetic variable, a complementary approach to identify genes within QTLs responsible for trait evolution could be to look for genotype–phenotype associations among France strains. We collected phenotype data from 51 France inbred lines with previously sequenced genomes (Lack et al., 2016) in order to perform genotype–phenotype association testing. This sample size would not be adequate for genome‐wide association testing, and so we restricted our focus to France QTL regions of less than 2 Mb in length. We performed this analysis either on all SNPs within these QTLs (120,243 SNPs), or focusing more specifically on SNPs within‐population genetic outliers windows (9480 SNPs). Genome‐wide significance, assessed via permutations, was not reached by any SNP in either analysis (Table S7). From the more inclusive analysis, the highest marker had a –log *p* value of 4.43, whereas the permutation significance threshold was 6.17. From the population genetic outlier analysis, the highest marker had a –log *p* value of 3.56 with permutation significance threshold of 4.44.

### 3.6 Simulation‐driven investigation of genetic architecture and association testing power

We then considered which genetic architectures our QTL mapping data might provide evidence for, and whether they might account for our negative association testing results. Although full model inference of adaptive evolution at the genetic level is beyond the scope of the present study, we conducted an exploratory simulation analysis in three stages, focusing on the two France crosses.

**FIGURE 5:**
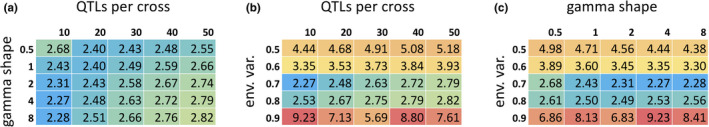
QTL simulations provide limited resolution on parameters underlying the evolution of ethanol resistance in the France population. Heat maps depict the mean relative error between empirical data (from France crosses) and selected simulated data sets, based on the QTL mapping summary statistics compared (see *Materials and Methods*). These plots each fix one of the three parameters with its value from the best‐matching parameter combination: (a) shows the case of an environmental variance of 0.7 (70% of individual trait variation explained by non‐genetic factors), (b) gamma shape parameter of 4 (i.e., shifted toward larger effect loci relative to an exponential distribution), and (c) 10 distinct QTLs present in each of the two crosses

First, we wanted to assess the number and strength of QTLs that our mapping data were most consistent with. Our simulations used a modified version of SIBSAM, which simulates the full mapping experiment (including recombination, phenotypic selection, and sequencing read sampling). We varied the number of QTLs present in each cross, their distribution of effect sizes as a function of the gamma distribution shape parameter, and the proportion of trait variation contributed by environmental/random effects rather than these QTLs. And we quantified properties of QTL peaks and genome‐wide ancestry in the simulated data and compared it with our empirical observations using mean relative error. The parameter combination with the lowest average mean relative error was 10 QTLs per cross, a gamma shape parameter of 4, and 70% environmental variation (Figure 5; Table S7). An otherwise identical parameter combination with a gamma shape parameter of 8 matched the empirical data almost equally as well, and so we chose an intermediate shape parameter of six in further analyses. Parameter combinations involving a wide range of QTL numbers and gamma shape values were non‐significantly worse than the above combination. Thus, larger data sets (especially with greater numbers of crosses) will be needed to make formal inferences of this type about the genetic architecture of a trait's adaptive evolution (Table S8).

Second, we assessed whether the degree of QTL overlap between the two France crosses provides information about the frequency of ethanol resistance alleles in this population. The QTL frequency that resulted in the average overlap of peaks that best matched the empirical data was 5% (Table S9), which resulted in ~54% overlap compared to the empirical ~44%. However, only the highest frequency values (90% and above) had confidence intervals that marginally excluded the empirical frequency. Here again, larger data sets with more independent crosses might help us to gain further resolution about the frequencies of adaptive variants.

**FIGURE 6:**
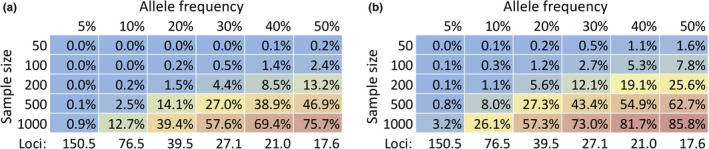
Larger sample sizes would be needed to perform powerful genotype–phenotype association within QTL regions, even if restricted to local adaptation outlier windows. Heat maps depict power to detect genotype–phenotype associations after multiple testing correction (the proportion of SNPs meeting an analysis‐wide significance threshold), as a function of the frequency of causative variants and the sample size of individuals/strains. These results are based on simulations (using a gamma shape parameter of six for the distribution of effect sizes) along with the *p* value thresholds identified from the empirical analysis. (a) corresponds to the scenario in which full QTL regions were tested, while (b) corresponds to the scenario in which only population genetic outlier windows within QTL regions were tested. The mean population‐wide numbers of loci that each simulation scenario required are also indicated below each panel

We therefore investigated a wide range of frequencies (5% to 50%) in assessing the power of our association testing analysis. The power analysis revealed that there is little power to detect causative SNPs that segregate at lower frequencies. Small‐to‐moderate population sizes (*n* = 50, 100, and 200) had low power to detect SNPs at any frequency (Figure 6). It was not until QTL frequency reached 50% that there was a greater than 10% detection power. Even when there was a large number of individuals used, for example, *n* = 1000, only ~0.9% of the causative SNPs at a 5% QTL frequency met the empirical threshold. Only once QTL frequency reached 30% was there greater than 50% detection rate. However, the detection power did improve when the lower outlier region threshold was used. The small population sizes had a greater than 10% detection power when QTL frequency reached 30%. The detection power at 5% QTL frequency for large population size of 1000 improved to ~3% and had a greater than 50% detection rate when QTL frequency reached 20%. Hence, significantly larger sample sizes would be needed to identify variants underlying polymorphic architectures of adaptive evolution, unless the number of suspected variants (and hence the multiple testing burden) could be further reduced.

## 4 DISCUSSION

We have shown that there is a range of ethanol resistance found in wild populations of *D*. *melanogaster*. The Zambia population, which inhabits the species’ ancestral range (Sprengelmeyer et al., 2020), is the least resistant with the recently diverged populations becoming more resistant. We show that elevated ethanol resistance is present not only at high latitudes, but also at high altitudes within Africa. When ethanol resistance for either a France, Ethiopia, or South Africa population is compared against Zambia, phenotypic differentiation (*Q*_ST_) strongly exceeds genetic differentiation (*F*_ST_)—suggesting that local adaptation has operated on this or a pleiotropically correlated trait. In agreement with other studies (David & Bocquet, 1975), we found the higher latitude France population to be highly resistant. Interestingly, *Aldh* (Fry et al., 2008) is contained in one of the France mapping crosses and *Adh* (Cohen & Graf, 1985) is contained in the other. However, only *Adh* has a population genetic signal of local adaptation based on our criteria. Both genes have been implicated in the latitudinal cline of increased ethanol resistance.

The BSA performed on the four different crosses revealed 32 significant QTLs with the largest estimated effect size for each cross between 12% and 27%. These data taken together suggest that ethanol resistance is moderately polygenic with moderate to large effect QTLs present (whereas smaller QTLs may elude our detection power; Pool, 2016). We found that there are no QTLs overlapping between all three high resistant populations. However, each of the high altitude populations shares QTLs with the France strains, whereas the two high altitude populations, South Africa and Ethiopia, do not have any QTLs in common with each other. Given the unequal levels of ethanol resistance between populations (Figure 1), some differences in genetic architecture would of course be expected.

In interpreting the observed levels of QTL overlap between populations, it is important to keep in mind that even between two crosses from the same resistant population (France), QTL overlap was modest. Of the seventeen significant QTL peaks between the two France crosses, they shared only six QTLs (and even some of these could reflect random overlap in light of the QTL sizes). For example, the strongest QTL in either France cross with an estimated effect size of 27% from FR305N on chromosome arm 3L is completely missing in FR364N. Our experiment should have very high power to detect a QTL with an effect size even roughly this large if it existed in a second cross (Pool, 2016). Those results suggest a genetically heterogeneous architecture of ethanol resistance evolution not only between populations but also within a resistant population. Notably, very similar patterns, both within and between populations, were also observed in similar experiments focused on the evolution of melanism within this species (Bastide et al., 2016). The implication that causative variants have not been fixed has multiple potential explanations, including ongoing adaptation, balancing selection, or that a trait has reached a new optimum value or exceeded a new threshold value. Persistent variability in the genetic basis of an adaptive trait might be expected when populations start with abundant standing genetic variation, as might be expected for *D*. *melanogaster*.

Still, our simulation results clearly show that larger experiments will be needed to gain quantitative resolution on key parameters that describe the genetic architecture of adaptive evolution. Studies with larger numbers of QTL mapping crosses may allow clearer estimation of the number of QTLs per cross, the distribution of QTL effect sizes, and the frequencies of causative variants in an evolved population. The utility of genotype–phenotype association testing will depend on either much larger population samples of sequenced inbred line genomes becoming available, or else further progress in restricting the number of SNPs to be tested. Candidate SNPs might be further limited by more precise QTL mapping (more generations, more individuals), functional genomic data, or complementary population genomic analysis such as genotype–environment association. Regardless of the strategy chosen, the results of this study and others suggest that future efforts to understand the genetic basis of adaptive trait evolution should allow for the likelihood of a genetically variable trait architecture among individuals such as that detected here.

## CONFLICT OF INTEREST

The authors declare that no conflict of interest exists.

## AUTHOR CONTRIBUTION

**Quentin D. Sprengelmeyer:** Conceptualization (supporting); Data curation (lead); Formal analysis (lead); Funding acquisition (supporting); Investigation (lead); Methodology (supporting); Project administration (supporting); Visualization (lead); Writing‐original draft (lead); Writing‐review & editing (supporting). **John E. Pool:** Conceptualization (lead); Formal analysis (supporting); Funding acquisition (lead); Investigation (supporting); Methodology (lead); Project administration (lead); Supervision (lead); Visualization (supporting); Writing‐original draft (supporting); Writing‐review & editing (lead).

## Supporting information

Table S1

Table S2

Table S3

Table S4

Table S5

Table S6

Table S7

Table S8

Table S9

## Data Availability

Raw sequence read data are available in the NIH SRA under accession number PRJNA686135.
